# AppleMDO: A Multi-Dimensional Omics Database for Apple Co-Expression Networks and Chromatin States

**DOI:** 10.3389/fpls.2019.01333

**Published:** 2019-10-22

**Authors:** Lingling Da, Yue Liu, Jiaotong Yang, Tian Tian, Jiajie She, Xuelian Ma, Wenying Xu, Zhen Su

**Affiliations:** State Key Laboratory of Plant Physiology and Biochemistry, College of Biological Sciences, China Agricultural University, Beijing, China

**Keywords:** *Malus domestica*, co-expression network, functional module, chromatin state, fruit ripening, anthocyanin biosynthesis

## Abstract

As an economically important crop, apple is one of the most cultivated fruit trees in temperate regions worldwide. Recently, a large number of high-quality transcriptomic and epigenomic datasets for apple were made available to the public, which could be helpful in inferring gene regulatory relationships and thus predicting gene function at the genome level. Through integration of the available apple genomic, transcriptomic, and epigenomic datasets, we constructed co-expression networks, identified functional modules, and predicted chromatin states. A total of 112 RNA-seq datasets were integrated to construct a global network and a conditional network (tissue-preferential network). Furthermore, a total of 1,076 functional modules with closely related gene sets were identified to assess the modularity of biological networks and further subjected to functional enrichment analysis. The results showed that the function of many modules was related to development, secondary metabolism, hormone response, and transcriptional regulation. Transcriptional regulation is closely related to epigenetic marks on chromatin. A total of 20 epigenomic datasets, which included ChIP-seq, DNase-seq, and DNA methylation analysis datasets, were integrated and used to classify chromatin states. Based on the ChromHMM algorithm, the genome was divided into 620,122 fragments, which were classified into 24 states according to the combination of epigenetic marks and enriched-feature regions. Finally, through the collaborative analysis of different omics datasets, the online database AppleMDO (http://bioinformatics.cau.edu.cn/AppleMDO/) was established for cross-referencing and the exploration of possible novel functions of apple genes. In addition, gene annotation information and functional support toolkits were also provided. Our database might be convenient for researchers to develop insights into the function of genes related to important agronomic traits and might serve as a reference for other fruit trees.

## Introduction

Apple (*Malus domestica* Borkh.), a member of the Rosaceae family, is one of the most cultivated fruit trees in temperate regions worldwide, and its origin and evolution are inseparable from the progress of human civilization (Duan et al., 2017). As an economically important crop, apple is rich in many nutrients, such as sugars, acids, aromatic alcohols, pectin substances, vitamins, and mineral elements, as well as flavonoids. In recent years, breeding methods and biotechnological strategies have been used to cultivate valuable apple cultivars for consumers’ preferences, such as color, flavor, and flesh texture. As important secondary metabolites, anthocyanins are not only pigment compounds responsible for colors in many fruits but also potential antioxidants that are beneficial for human health (Dixon et al., 2005). It was reported that some genes (*MdJAZ18*, *MdSnRK1*.1, *MdMYB9*, *MdMYB11*, *MdTTG1*, *MdBBX20*, etc.) regulate anthocyanin biosynthesis (Brueggemann et al., 2010; An et al., 2015; Liu et al., 2017; Fang et al., 2019). Large amounts of volatile esters accumulated in apple contribute characteristic fruity notes. Several genes have been reported to regulate volatile esters, such as *MdACS3*, *MdAAT1*, *MdPG1*, *MdADH*, and *MdSDR* (Farneti et al., 2017). Ethylene, an important plant hormone, regulates several physiological processes of fruit ripening (Costa et al., 2005), which is closely related to the long-distance transport and shelf life of apple (Costa et al., 2005). It has been confirmed in tomato that ethylene level has a direct relationship with fruit softening (Rose et al., 1997). Since 2010, the whole genomes of *M. domestica* cv. “Golden Delicious” (Velasco et al., 2010; Li et al., 2016; Daccord et al., 2017) and “Hanfu” (Zhang et al., 2019) were sequenced and reported. Following the success of whole-genome sequencing of apple, research on the molecular biology of apples has progressed rapidly. Molecular marker-assisted breeding is gradually applied to accelerate the apple breeding process. However, there are still many genes with unknown functions in apple, which pose a great challenge for cultivating valuable apple varieties. Recently, it was reported that many omics datasets had been used for the prediction of the expected breeding values of agronomic traits (Wang et al., 2019; Frisch et al., 2010). An integrated analysis of various omics datasets has the potential to advance our knowledge of the underlying genetic mechanisms of important agronomic traits.

With the development of sequencing technologies, a large number of transcriptomic datasets for apple have accumulated, which include datasets for various tissues, developmental stages, and stress treatments. Gene co-expression networks are network diagrams based on the similarity of expression levels between genes. At present, co-expression networks are widely applied to many animals and plants, such as COXPRESSdb v7 (http://coxpresdb.jp) for 11 model animals (Obayashi et al., 2019), ATTED-II (http://atted.jp/) and PlaNet (http://aranet.mpimp-golm.mpg.de/) for several plants (Mutwil et al., 2011; Obayashi et al., 2018), ccNet (http://structuralbiology.cau.edu.cn/gossypium/) (You et al., 2017) for cotton, MCENet (http://bioinformatics.cau.edu.cn/MCENet/) for maize (Tian et al., 2017), WheatNet (www.inetbio.org/wheatnet) for wheat (Lee et al., 2017), VTCdb (http://vtcdb.adelaide.edu.au/home.aspx) for grape (Wong et al., 2013), and so on. At present, the accumulation of transcriptomic datasets also makes it possible to construct co-expression networks for apple.

It has been reported that many different epigenetic modifications exist simultaneously in the same part of the genome, which indicates that epigenetic modification occurs synergistically in multiple dimensions (Strahl and Allis, 2000). The method of characterizing a variety of different epigenetic markers into chromatin states has been applied in animals and plants (Ernst and Kellis, 2012). A variety of epigenomic profiles of different epigenetic markers have been produced for apple, using DNase-seq, ChIP-seq, and Bisulfite-seq. These datasets can be used to identify potential regulatory elements in the genome at the whole-genome level. Currently, the fruitENCODE database (http://137.189.43.55/encode.html) provides a genome browser for a variety of fruits, including apple, to view DNA methylation, DNase I hypersensitivity sites (DHSs), and histone modification (Lu et al., 2018). The Genome Database for Rosaceae (https://www.rosaceae.org/) is a popular genome database for Rosaceae that provides genomic, genetic, and breeding data (Jung et al., 2019).

Whole-genome transcriptome and epigenome analyses are useful approaches for predicting genes with biological functions. However, there is currently no integrated platform for fruit transcriptomic and epigenomic datasets, and information mining by integrated analysis is lacking compared with that in *Arabidopsis* (Liu et al., 2018; Obayashi et al., 2018). It is urgent to effectively use a large number of high-throughput sequencing datasets for apple. Thus, we developed a multi-dimensional omics database for apple co-expression networks and chromatin states (AppleMDO), which will help in the cross-referencing and exploration of some novel functions of genes and provide a reference for other fruits.

## Material and Methods

### RNA-Seq Data Procession

The raw reads of RNA-seq datasets were filtered with FastQC (version 0.11.2) (https://www.bioinformatics.babraham.ac.uk/projects/fastqc/), and low-quality reads were removed by FASTX Toolkit (version 0.0.13) (http://hannonlab.cshl.edu/fastx_toolkit/). Cutadapt (version 1.8.3) (http://cutadapt.readthedocs.io/en/stable/) was used to remove adaptor sequences. The clean RNA-seq data were aligned to the reference genome (GDDH13 version 1.1) (https://iris.angers.inra.fr/gddh13/) by using TopHat (version 2.0.9) (Trapnell et al., 2009), and fragments per kilobase per million fragments mapped (FPKM) values were calculated using Cuffdiff (version 2.2.1) (Trapnell et al., 2010). Then, the outlier samples were excluded through a cluster analysis performed on all datasets with the R package “pheatmap” (version 1.0.8) (https://cran.r-project.org/src/contrib/Archive/pheatmap/) (**Supplementary Figure 1**).

### Co-Expression Network Construction

Pearson correlation coefficients (PCCs) were calculated to quantify the correlations between genes. Then, we screened for highly correlated gene pairs based on the ranking of PCC values by mutual rank (MR) algorithms. The calculation formulas for PCCs and MR are as follows:

$$\text{PCC}=\frac{\sum \left(X-\bar{X})(Y-\bar{Y}\right)}{\sqrt{\sum_{i=1}^{n}{\left(X_{i}-\bar{X}\right)}^{2}}\sqrt{\sum_{i=1}^{n}{\left(Y_{i}-\bar{Y}\right)}^{2}}}$$

$$\text{MR}(\text{AB})=\sqrt{\mathrm{Rank}(A\to B)\times \mathrm{Rank}(B\to A)}$$

where x and y are FPKM values, n is the total number of samples, and Rank(A→B) represents PCC ranking of gene A in all co-expression genes with gene B.

Furthermore, biological process gene ontology (GO) terms associated with a number of genes in the interval [4, 20] were selected as the prior knowledge to measure the accuracy of the co-expression network by the area under the ROC curve (You et al., 2017; Tian et al., 2017). By comparing area under the ROC curve values under different thresholds, the optimal PCC and MR values were selected as thresholds to construct the co-expression network.

### Module Identification and Annotation

The clique percolation method locates the k-clique percolation clusters of the network, which we interpreted as modules (Derenyi et al., 2005). CFinder software (version 2.0.6) (Adamcsek et al., 2006) was used to identify modules in the apple co-expression network. When k = 6 cliques, there is a greater number of functional modules (communities), more gene coverage, and more community overlap (**Supplementary Figure 4**). Functional annotation of the module was predicted by gene set enrichment analysis, which referred to PlantGSEA (Yi et al., 2013). Significant entries were reserved based on Fisher’s test and multiple hypothesis testing (FDR ≤ 0.05).

### Chromatin State Definition

After quality filtering and adaptor removal with FastQC (version 0.11.2) (https://www.bioinformatics.babraham.ac.uk/projects/fastqc/) and Cutadapt (version 1.8.3) (http://cutadapt.readthedocs.io/en/stable/), the clean reads from epigenomic datasets were aligned to the reference genome (GDDH13 version 1.1) (https://iris.angers.inra.fr/gddh13/) by Bowtie2 (version 4.1.2) (Langmead and Salzberg, 2012) with default parameters. Then, model-based analysis of ChIP-Seq (version 1.4.1) (Zhang et al., 2008) was used to call peaks with default parameters. Cis-regulatory Element Annotation System (version 1.0.2) (Shin et al., 2009) was used to calculate the positional distribution of the epigenetic marks on the genome. plotCorrelation in deepTools software (version 2.2.4) was used to calculate correlations based on normalized wig files, and outlier samples were excluded, confirming that the same types of epigenetic datasets were clustered together (**Supplementary Figure 7**). ChromHMM (version 1.12) (Ernst and Kellis, 2012), based on a multivariate hidden Markov model (HMM), was used to model the binary presence or absence of each chromatin mark in 200-bp bins over the whole genome. LearnModel of ChromHMM was used to learn from binarized data and divide the genome into 200-bp segments, and the numstates parameter was initially set as 10 to 50. CompareModels in ChromHMM was applied to compare all learned models with the 50-states model to choose the best model according to similarity. OverlapEnrichment in ChromHMM was applied to analyze fold enrichments of chromatin states relative to epigenetic modifications and the genomic-feature regions (promoters, 5’ untranslated regions, exons, introns, 3’ untranslated regions, intergenic regions and transposable elements) (Ernst and Kellis, 2010; Ernst et al., 2011; Baker et al., 2015; Liu et al., 2018).

### Gene Family Identification

Transcription factors and protein kinase families were identified by iTAK software (http://bioinfo.bti.cornell.edu/cgi-bin/itak/index.cgi) (Perez-Rodriguez et al., 2010). For transcription factors, some special Pfam domains were also considered; for example, AUX/IAA family members contain only one PF02309 domain, and PF06507 and PF02362 domains are prohibited. Ubiquitin families were identified by a hidden Markov model obtained from UUCD (http://uucd.biocuckoo.org/) (Gao et al., 2013). The carbohydrate-active enzyme families and epigenetic regulators were obtained based on orthologous genes in *Arabidopsis thaliana* predicted by InParanoid (version 4.1) (http://inparanoid.sbc.su.se/cgi-bin/index.cgi) (Remm et al., 2001; O’Brien et al., 2005; Sonnhammer and Ostlund, 2015) (bootstrap ≥ 0.6) software and Pfam domains. For the CYP450 family, we provided 346 CYP450 members by blasting with 348 members of the v.10 genome collected from the Cytochrome P450 database (http://drnelson.uthsc.edu/CytochromeP450.html).

### Motif Analysis

A total of 1,035 motifs were collected from several publications (Bolduc et al., 2012; Ramireddy et al., 2013; Franco-Zorrilla et al., 2014) and public databases PLACE (Higo et al., 1999), PlantCARE (Lescot et al., 2002), and AthaMap (Hehl and Bulow, 2014). Significantly enriched motifs can be identified by scanning for these motifs in the promoter sequences of submitted genes based on Z-scores and P-values (You et al., 2017; Liu et al., 2018). The calculation formulas for the Z-scores and P-values are as follows:

$$\text{Z-score}=\frac{N_{\mathrm{motif}}-{\mathrm{mean}}_{\mathrm{motif}}}{{\mathrm{stdev}}_{\mathrm{motif}}}$$

$$\text{P-value}=1-\text{pnorm}(N_{\mathrm{motif}},{\mathrm{mean}}_{\mathrm{motif}},{\mathrm{stdev}}_{\mathrm{motif}},)$$

*N_motif_* represents the number of occurrences of a motif in 3,000-bp promoters of the genes submitted, *mean_motif_* represents the average number of occurrences of the motif in the background (the 3,000-bp promoter of *m* genes randomly selected 1,000 times), and *stdev_motif_* corresponds to the *mean_motif_*.

### Analysis Tools

GO analysis: GO analysis was used to find significantly enriched GO terms for gene of interest based on GO annotation obtained by BLAST (version 2.2.19) and Blast2GO, which referred to agriGOv2 (Tian et al., 2017). ID conversion: Gene ID conversion was performed for different species by InParanoid (version 4.1) (http://inparanoid.sbc.su.se/cgi-bin/index.cgi) (Remm et al., 2001; O’Brien et al., 2005; Sonnhammer and Ostlund, 2015) (bootstrap ≥ 0.6) based on protein sequences and for two genome versions of apple by BLAST (version 2.2.19) based on nucleotide sequences. Sequence extraction: The gene sequences were extracted based on the gene IDs or the positions of the genes in the genome. University of California Santa Cruz (UCSC) genome browser: Combined with the gene structure information, the alignment results for the transcriptomic and epigenomic datasets were uploaded to the UCSC genome browser to visually display the expression profiles and histone modifications of genes (Haeussler et al., 2019).

Orthologue identification: The orthologues of apple genes in 13 species (*A. thaliana, Prunus persica, Pyrus communis, Pyrus x bretschneideri, Rosa multiflora, Rubus occidentalis, Fragaria vesca, Vitis vinifera, Solanum lycopersicum, Populus Trichocarpa, Nicotiana benthamiana, Oryza sativa, and Zea mays*) were predicted by InParanoid (version 4.1) (http://inparanoid.sbc.su.se/cgi-bin/index.cgi) (Remm et al., 2001; O’Brien et al., 2005; Sonnhammer and Ostlund, 2015) with bootstrap ≥ 0.6.

Pfam domain: Conserved domains in protein sequences were predicted using PfamScan (https://www.ebi.ac.uk/Tools/pfa/pfamscan/) based on multiple sequence alignments and a hidden Markov model (Finn et al., 2016).

### Search and Visualization Platform

The AppleMDO database is supported by Red Hat Linux, Apache server (https://www.apache.org/), MySQL (https://www.mysql.com/), and PHP (https://php.net/) scripts. The visualization of the network was implemented in Cytoscape.js (http://js.cytoscape.org/) (Franz et al., 2016), which is an open source JavaScript package.

## Database Contents

### Data Resources

With a multi-dimensional omics perspective, many datasets were integrated to construct the AppleMDO database, including genomic, transcriptomic, and epigenomic datasets. The reference genome was GDDH13 version 1.1 from The Apple Genome and Epigenome database (https://iris.angers.inra.fr/gddh13/), which contains 45,116 protein-coding genes (Daccord et al., 2017). Transcriptomic datasets (RNA-seq) and epigenomic datasets (ChIP-seq, DNase-seq, and BS-seq) of “Golden Delicious” apple were collected from the National Center for Biotechnology Information Gene Expression Omnibus (http://www.ncbi.nlm.nih.gov/geo/) (Barrett et al., 2013) and Sequence Read Archive (http://www.ncbi.nlm.nih.gov/sra) (Kodama et al., 2012). Compared with the “Golden Delicious” variety, the datasets of other varieties are mainly for limited tissues and developmental stages, although a large number of transcriptomic datasets have accumulated in public databases covering many apple varieties. All the publicly available epigenomic datasets are for the “Golden Delicious” variety. More importantly, the “Golden Delicious” variety has a complete genome sequence, so the datasets of the “Golden Delicious” variety were selected for subsequent analysis.

A total of 112 transcriptomic datasets were collected, including those for various tissues (seedling, bud, flower, fruit, seed, shoot apex, stem, cotyledon, and leaf) and stress treatments (pathogen infection). In more detail, there were several datasets for different growth stages of tissues, for example, flower bud datasets from dormancy to germination, fruit flesh datasets collected at different weeks after full bloom, and datasets for various floral organs (**Table 1**). These datasets are comprehensive and detailed and reflect the gene expression patterns to a great extent.

**Table 1:** RNA-seq data resources.

Tissue	Sample information	Experiment	Reference
seedling	seedling	SRR768136	INRA
bud	bud break; dormant buds (0/1/2/3/4 months)	SRP099578	Foundation Edmund Mach
flower	mature	SRS1558530	PMID: 27503335 (Li et al., 2016)
stigmas	open flowers	SRR6308190	IBMC/i3S
styles	open flowers	SRR6308181	
filaments	open flowers	SRR6308188	
anthers	1-3 days prior to flower opening	SRR6308187	
petals	open flowers	SRR6308191	
pollen	open flowers	SRR6308192	
sepals	open flowers	SRR6308194	
receptacles	open flowers	SRR6308193	
ovaries	open flowers	SRR6308189	
fruit	1-20 WAFB	SRR3384922	PMID: 25576355 (Bai et al., 2015)
	25/35/60/87 DPA	SRP018878	INRA
	immature/mature	SRP102870	PMID: 30250279 (Lu et al., 2018)
	mature, mock/CreA/PhleoR infected with *P. expansum*	SRP150975	PMID: 30047230 (Tannous et al., 2018)
fruit peel	mature	SRP102870	PMID: 30250279 (Lu et al., 2018)
seed	20 DAPF	SRP048976	PMID: 25781174 (Ferrero et al., 2015)
shoot apex	4-6-week-old seedling	SRX765691	Michigan State University
	new shoot	SRX765683	
stem	mature	SRS1558540	PMID: 27503335 (Li et al., 2016)
cotyledon	mock/pale green lethal seedling	SRP069858	https://link.springer.com/article/10.1007/s11295-016-1097-5
leaf	plantlets, mock/ASGV-infected	SRP034943	PMID: 24736405 (Chen et al., 2014)
	fully developed, 0-14 DPA infected with *V. inaequalis*	SRP018878	INRA
	immature	SRR6308182	IBMC/i3S
	youngest/oldest leaf; mock/infected with *V. inaequalis*_72/96 h	ERP003589	PMID: 24223809 (Gusberti et al., 2013)
	mature	SRS1206445	PMID: 27503335 (Li et al., 2016)
	mature	SRP102870	PMID: 30250279 (Lu et al., 2018)

Additionally, 20 epigenomic datasets were collected, which included histone modification (H3K4me3, H3K27me3, and H3K36me2), DNase-seq, and Bisulfite-seq datasets (**Table 2**). We considered the activation and repression of transcriptional regulation by epigenetic marks, for example, DHSs and H3K4me3 as activation marks and H3K27me3 and DNA methylation as inhibition marks. In addition, the different dominant positions of epigenetic marks, for example, DHSs in the promoter region, H3K4me3 downstream of the TSS region, and H3K27me3 and H3K36me2 in the entire gene body region, were as comprehensive as possible.

**Table 2:** Epigenomic data resources.

Type	Tissue	Sample information	SRA experiment	Reference
DNase-seq	leaf	mature	SRX2697891, SRX2697892	PMID: 30250279 (Lu et al., 2018)
	fruit flesh	immature	SRX3420379, SRX3420380, SRX3420381	
	fruit flesh	mature	SRX2697889, SRX2697890	
H3K27me3	leaf	mature	SRX768318	Michigan State University
	leaf	mature	SRX2697980, SRX2697981	PMID: 30250279 (Lu et al., 2018)
	fruit flesh	immature	SRX3420335	
	fruit flesh	mature	SRX2697978, SRX2697979	
	shoot apex	new shoot (6-10 leaves)	SRX768312	Michigan State University
H3K4me3	leaf	new shoot (6-10 leaves)	SRX768320	
	shoot apex	new shoot (6-10 leaves)	SRX768315	
H3K36me2	leaf	new shoot (6-10 leaves)	SRX768319	
	shoot apex	new shoot (6-10 leaves)	SRX768314	
Bisulfite-seq	fruit	3 days after pollination	SRX2511185	PMID: 28581499 (Daccord et al., 2017)
	fruit	9 days after pollination	SRX2511186	

### Co-Expression Network Construction and Functional Module Identification Based on Transcriptomic Data

**Figure 1:**
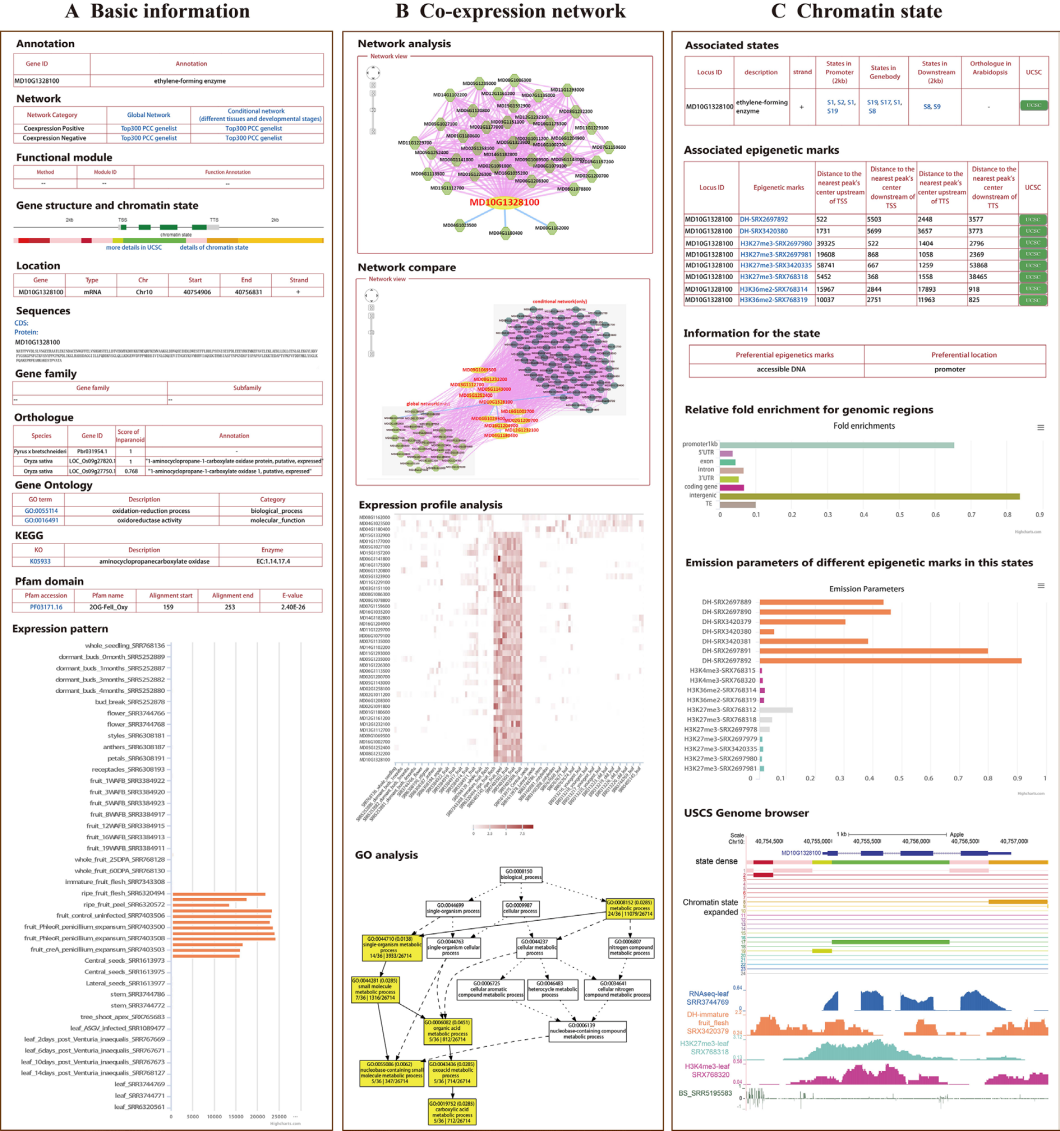
General description of AppleMDO functions. **(A)** Basic information for *MdACO1* obtained by using the search function in AppleMDO, including annotation, co-expression network, gene structure, chromatin states (with warm colors representing activation of transcription and cool colors representing inhibition of transcription), gene location, CDSs and proteins, gene family, orthologous genes in 13 species, gene ontology, Kyoto Encyclopedia of Genes and Genomes, Pfam domain, and expression patterns in various tissues and under different stresses. **(B)** Co-expression network analysis of *MdACO1*. A global or tissue-preferential co-expression network of *MdACO1* was found by searching for *MdACO1* (MD10G1328100) in an AppleMDO network analysis, including positive co-expression relationships, negative co-expression relationships, and possible protein–protein interactions. Further network comparison analysis has been implemented between the global network and tissue-preferential network. Moreover, for all genes in the network, we provide a GO enrichment analysis tool to further annotate gene functions and expression profile analysis tools to visualize gene expression levels. **(C)** Chromatin state analysis of *MdACO1*. Associated states and epigenetic marks of genes were found by searching for a single gene or a list of genes in gene analysis. Relative fold enrichment for genomic regions and emission parameters of different epigenetic marks in the state were found by searching for the state in state analysis. Furthermore, detailed information on states and the signals of epigenetic markers can be visualized in the genome using the UCSC genome browser.

All 112 RNA-seq datasets of “Golden Delicious” apple were integrated to construct a global network, including different tissues, developmental stages, and stress treatments, in order to analyze possible gene function correlations through gene expression similarities. In addition to the global network, we constructed a conditional network (tissue-preferential network) for 81 samples without stress treatment (**Table 1**). To measure the expression correlation between genes, PCC values were calculated; gene pairs with a PCC value in the interval (0.5, 1) were considered positively correlated, while those with a PCC value in the interval (-1, -0.3) were considered negatively correlated for both the global network and the tissue-preferential network (**Supplementary Figure 2**). Furthermore, strict parameters were set to filter co-expression gene pairs in order to increase the credibility of the co-expression relationships. After evaluation, the PCC and MR thresholds were determined for the global co-expression network (PCC ≥ 0.8 and MR ≤ 55) (**Supplementary Figure 3**), which included 97.2% (43,862/45,116) of the coding genes (**Supplementary Table 1**). The tissue-preferential co-expression network included 95.3% (42,991/45,116) (**Supplementary Figure 3**) of the coding genes, with a PCC ≥ 0.8 and an MR ≤ 50 (**Supplementary Table 1**). In AppleMDO, a search function for one gene or a list of genes was provided for the global and tissue-preferential co-expression networks, which were visualized by the Cytoscape web tool. Further network comparison analysis was implemented between the global network and the tissue-preferential network. For all genes in the network, we provided a GO enrichment analysis tool to further exploit functions and expression profile analysis tools in order to visualize expression levels (**Figure 1B**).

In addition, a total of 1,076 functional modules were identified to assess the modularity of the apple co-expression networks based on the clique percolation method algorithm, with more than or equal to six genes per module. Gene set enrichment analysis showed that the function of many modules was related to development, secondary metabolism, hormone response, and transcriptional regulation (**Supplementary Figure 5**).

### Chromatin State Analysis Based on Epigenomic Data

A single epigenomic dataset can reflect the distribution of only one epigenetic mark in the genome, but chromatin states are affected synergistically by a variety of epigenetic marks. Several platforms are reported to predict chromatin states through integrated analysis of epigenomic datasets in plants (Liu et al., 2018; Tian et al., 2017). A total of 20 epigenomic datasets, including histone modification datasets (H3K4me3, H3K27me3, and H3K36me2), DNase-seq datasets, and DNA methylation datasets, of “Golden Delicious” apple were integrated and used to classify chromatin states (**Table 2**). Based on the ChromHMM algorithm, the genome was divided into 620,122 fragments, which were classified into 24 states according to the combination of epigenetic marks and enriched-feature regions (**Table 3**, **Supplementary Figure 6**). Each state was marked in a different color according to the reported function of the epigenetic marks to reflect transcriptional activity, in which the states with activation of transcription were marked in warm colors and the states with inhibition of transcription were marked in cool colors (**Supplementary Figure 8**). For example, state 2 was marked in red because accessible DNA is its preferential epigenetic mark and promoters and intergenic regions are its preferential positions. In AppleMDO, the chromatin states of the genes and the epigenome markers of states can be searched, and the sign of epigenome markers at each gene or state can be visualized by the UCSC genome browser (**Figure 1C**).

**Table 3:** AppleMDO content.

Database content		Detailed information	Method
Network	Global network	43,862 genes (759,862 edges)	PCC & MR
	Tissue-preferential network	42,991 genes (683,265 edges)	PCC & MR
	Protein-protein interaction	7,298 genes (37,406 edges)	InParanoid
Module	Functional module	9,133 genes (1,075 modules)	CFinder
Chromatin state	Chromatin state	24 states (620,122 segments)	HMM
Gene family	Cytochrome P450	346 genes (88 families)	Blast & InterProScan
	Protein kinase	1,991 genes (87 families)	iTAK
	Ubiquitin	1,306 genes (20 families)	HMM
	Transcription factor/regulator	2,965 genes (83 families)	iTAK
	Carbohydrate-active enzyme	1,048 genes (94 families)	InParanoid
	Epigenetic regulator	822 genes (113 families)	InParanoid
Annotation	GO annotation	26,714 genes (65,061 entries)	Blast2GO
	KEGG annotation	10,343 genes (2,910 entries)	Orthologue
	Pfam domain	33,445 genes (55,187 domains)	PfamScan
	Orthologues in *A. thaliana*	18,838 genes (26,028 pairs)	InParanoid
	Orthologues in *P. persica*	19,110 genes (30,789 pairs)	InParanoid
	Orthologues in *P. communis*	23,333 genes (24,252 pairs)	InParanoid
	Orthologues in *P. bretschneideri*	21,758 genes (25,038 pairs)	InParanoid
	Orthologues in *R. multiflora*	20,256 genes (25,606 pairs)	InParanoid
	Orthologues in *R. occidentalis*	19,445 genes (20,055 pairs)	InParanoid
	Orthologues in *F. vesca*	19,093 genes (19,558 pairs)	InParanoid
	Orthologues in *V. vinifera*	18,995 genes (20,019 pairs)	InParanoid
	Orthologues in *S. lycopersicum*	14,503 genes (19,912 pairs)	InParanoid
	Orthologues in *P. trichocarpa*	22,030 genes (28,549 pairs)	InParanoid
	Orthologues in *N. benthamiana*	20,979 genes (31,248 pairs)	InParanoid
	Orthologues in *O. sativa*	19,683 genes (29,722 pairs)	InParanoid
	Orthologues in *Z. mays*	16,908 genes (21,785 pairs)	InParanoid

### Functional Annotations

At present, the vast majority of apple gene functions are unknown, so some functional annotations and structural annotations of genes are provided in AppleMDO. These gene functional annotations included gene family classification, gene ontologies, protein–protein interactions, and orthologous genes in other species. We classified 346 genes in 88 CYP450 families, 1,991 genes in 87 protein kinase families, 1,306 genes in 20 ubiquitin families, 2,965 genes in 83 transcription factor or regulator families, 1,048 genes in 94 carbohydrate-active enzyme families, and 822 genes in 113 epigenetic regulator families (**Table 3**). GO annotations of 26,714 genes were obtained by reference to agriGOv2 (**Table 3**). The annotation of KEGG pathways included 10,343 genes, which were downloaded from the Genome Database for Rosaceae (https://www.rosaceae.org/). Orthologous genes in 13 other species were also provided. *P. bretschneideri*, *P. communis*, *P. persica*, *F. vesca*, *R. multiflora*, and *R. occidentalis* are members of the Rosaceae family. *A. thaliana*, the most common model plant, is currently the most fully annotated dicotyledonous species. *S. lycopersicum*, a model plant for horticultural crops, is widely researched. *V. vinifera* was the first fruit with a complete genome sequence and is used in rootstock breeding. *P. trichocarpa* is a model plant for woody plants. *N. benthamiana* is an important model crop in plant pathology. *O. sativa* and *Z. mays* are important food crops and are widely studied. These species are representative in various ways and may be helpful in studying the functions of unknown genes in apple. The structural annotations included Pfam domains and images of gene structure for every gene (**Table 3**). In addition, gene expression profiling was performed to determine the expression level of each gene in different tissues, at different growth stages, and under different stress treatments based on transcriptomic datasets (**Figure 1A**).

Moreover, protein–protein interactions of *A. thaliana* and *O. sativa* were integrated from databases (Xenarios et al., 2002; Licata et al., 2012; Patel et al., 2012; Orchard et al., 2014; Reiser et al., 2016; Oughtred et al., 2019) and the literature (Lumba et al., 2014). As a result, 37,406 possible protein–protein interactions for apple were obtained based on orthologous genes in *A. thaliana* and *O. sativa*.

### Functional Support Tools

In AppleMDO, several analysis tools, including gene ontology enrichment analysis, blast analysis, motif analysis, ID conversion, sequence extraction, and the UCSC genome browser, are provided for users to conveniently explore potential functions of apple genes.

## Case Study

### Co-Expression Network Analysis of Fruit Ripening-Related Genes

**Figure 2:**
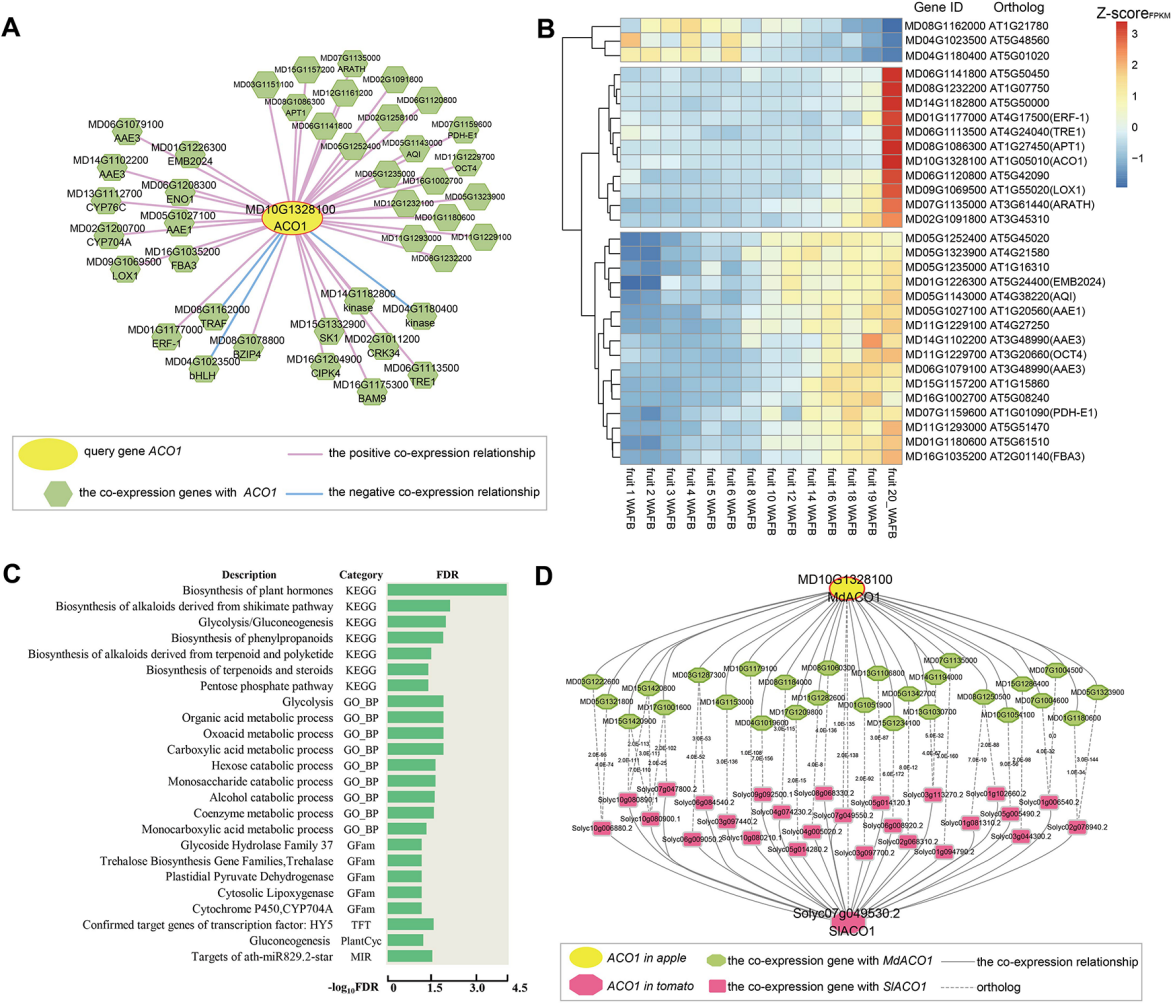
Global co-expression network analysis of *MdACO1*, associated with fruit ripening. **(A)** The global co-expression network of *MdACO1* (MD10G1328100). The middle yellow circle is the *ACO1* gene, and the surrounding green polygons are genes co-expressed with *MdACO1*. The red line indicates positive co-expression with *MdACO1*, and the blue line indicates negative co-expression with *MdACO1*. **(B)** Heatmap of all genes in the global co-expression network for *MdACO1*. The redder the color is, the higher the expression level is, and the bluer the color is, the lower the expression level is. **(C)** Gene set enrichment analysis of all genes in the global co-expression network for *MdACO1* by PlantGSEA (http://structuralbiology.cau.edu.cn/PlantGSEA). **(D)** Comparison of the top 300 genes in the global positive co-expression network between *MdACO1* in apple and *SIACO1* in tomato.

The ripening and softening of apple fruit are controlled by ethylene (Chagne et al., 2014). 1-aminocyclopropane-1-carboxylate oxydase (ACO1), a key gene involved in ethylene biosynthesis, oxidizes *ACC* to synthesize ethylene. It has been reported that the *ACO1* gene is related to the amount of ethylene released and the hardness of the fruit and plays an important role in the ripening process of apple fruit (Costa et al., 2005; Binnie and McManus, 2009). The global co-expression network of *MdACO1* was found by performing a network analysis of *MdACO1* (MD10G1328100) in AppleMDO, including positive and negative co-expression relationships (**Figure 2A**). As shown in **Figure 2A**, there are many genes related to fruit ripening in the co-expression network of *MdACO1*, such as *ENO1* (MD06G1208300), *LOX1* (MD09G1069500), and *CYP76C4* (MD13G1112700). It has been reported that LOX is related to ethylene content and contributes to aroma and flavor generation during fruit development in tomato (Griffiths et al., 1999), and the activity of LOX increases during the ripening and softening processes of kiwifruit and peaches (Zhang et al., 2003; Han et al., 2011). In addition, some transcription factors, for example, *ERF* (MD01G1177000) and *bHLH* (MD04G1023500), and protein kinases are included in the network of *MdACO1*. Wang et al. (2007) found that *MdERF* (MD01G1177000) is involved in apple fruit ripening (Wang et al., 2007). Through co-expression network analysis, genes with relative functions were found, suggesting that we can use this method to explore possible regulatory mechanisms of genes.

We also found that the expression patterns of these co-expressed genes were highly similar and that the activity of positively co-expressed genes was significantly higher in mature fruit samples than in other tissue samples based on cluster analysis of the expression profiles of *ACO1* co-expressed genes. However, the expression pattern of three genes that were negatively co-expressed with ACO1 was the exact opposite (**Supplementary Figure 9**). Interestingly, we found that the expression levels of some genes gradually increase as the fruit matures, for example, *ERF* (MD01G1177000), *bHLH* (MD04G1023500), and *LOX* (MD09G1069500), but those of other genes increase rapidly during the late stage of fruit ripening, for example, *AAE* (MD05G1027100, MD14G1102200, and MD06G1079100), *FBA3* (MD16G1035200), and *AQI* (MD05G1143000). In contrast, the expression levels of these three negatively co-expressed genes gradually decreased with fruit ripening (**Figure 2B**). Furthermore, gene set enrichment analysis of genes co-expressed with *ACO1* showed that these genes are mainly related to the biosynthesis of plant hormones, the biosynthesis of alkaloids and steroids, glycolysis, the alcohol catabolic process, and the biosynthesis of phenylpropanoids (**Figure 2C**). Therefore, we could predict the regulatory function of one gene involved by analysing its co-expression network.

Furthermore, we compared the co-expression networks of *ACO1* between different species. By comparing the top 300 genes in the positive co-expression network of *ACO1* between apple and tomato, we found that many genes in the two networks were orthologous, including *ACO2* (ACC oxidase 2), *LOX* (lipoxygenase), *AAE1* (acyl activating enzyme 1), *SDRd* (short-chain dehydrogenase/reductase isoform d), and *NAC2* (NAC domain containing protein 2), indicating that the genes in the *ACO1* co-expression network are not only different between species but also conserved (**Figure 2D**).

### Application of a Co-Expression Network in the Anthocyanin Biosynthesis Pathway

**Figure 3:**
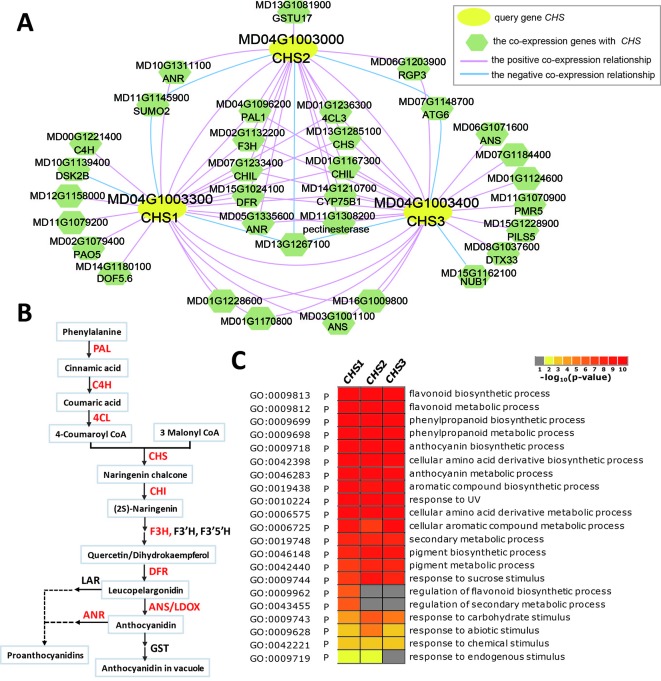
Global co-expression network analysis of *MdCHS*, related to anthocyanin biosynthesis. **(A)** The global co-expression network of *MdCHS1* (MD04G1003300), *MdCHS2* (MD04G1003000), and *MdCHS3* (MD04G1003400). The yellow circles are the three *MdCHS* genes, and the green polygons are genes co-expressed with *MdCHS*. The red line indicates positive co-expression with *MdCHS*, and the blue line indicates negative co-expression with *MdCHS*. **(B)** Anthocyanin biosynthesis pathway. The genes marked in red are in the global co-expression network of *MdCHS*. **(C)** GO enrichment analysis of all genes in the global co-expression network for *MdCHS1*, *MdCHS2*, and *MdCHS3* by agriGOv2 (http://systemsbiology.cau.edu.cn/agriGOv2/).

Due to its contribution to apple color and nutrition, anthocyanin biosynthesis in apples has been the subject of much research. Anthocyanin biosynthesis is somewhat conserved among species, and many structural genes (*PAL*, *C4H*, *4CL*, *CHS*, *CHI*, *F3H*, *DFR*, *ANS*/*LDOX*, and *UFGT*) involved in anthocyanin biosynthesis and some transcription factors (MYB-bHLH-WD40 complex, *WRKY*, and *BBX*) that regulate the expression of structural genes have been characterized in fruit plants, including apple, peach, Chinese pear, and European pear (Kim et al., 2003; Takos et al., 2006; Xie et al., 2011; Jaakola, 2013; Vrancken et al., 2013; El-Sharkawy et al., 2015; Yahyaa et al., 2017; Wang et al., 2018; Fang et al., 2019). Chalcone synthase (CHS), a key enzyme involved in the biosynthesis of flavonoids, catalyses 4-coumaroyl CoA and malonyl CoA to produce naringenin chalcone. Three chalcone synthases (*CHS1*: MD04G1003300, *CHS2*: MD04G1003000, and *CHS3*: MD04G1003400) were identified in apple leaves (Yahyaa et al., 2017). When three *CHS* gene IDs (MD04G1003300, MD04G1003000, and MD04G1003400) are entered into the search box of the network analysis in AppleMDO, their co-expression networks are obtained (**Figure 3A**). The co-expression networks of *CHS1*, *CHS2*, and *CHS3* are highly intersected, with 11 genes shared by the three networks and 8 genes shared by two networks, and most of the co-expressed genes are involved in the anthocyanin biosynthesis pathway, such as *PAL1* (MD04G1096200), *C4H* (MD00G1221400), *4CL3* (MD01G1236300), *CHI* (MD01G1118000 and MD01G1118100), *CHIL* (MD07G1233400 and MD01G1167300), *F3H* (MD02G1132200), *DRF* (MD15G1024100), *ANS*/*LDOX* (MD03G1001100 and MD06G1071600), and *ANR* (MD10G1311100) (**Figures 3A, B**). Furthermore, GO enrichment analysis was performed on the co-expressed genes of *CHS1*, *CHS2*, and *CHS3* using agriGOv2 and showed that these genes were related to the flavonoid biosynthetic process, phenylpropanoid biosynthetic process, anthocyanin biosynthetic process, and secondary metabolic process, which demonstrated that the network of *MdCHS* corresponded to the anthocyanin biosynthetic pathway and that network analysis helped improve the functional annotation of *MdCHS* in apple (**Figure 3C**).

### Further Analysis of Three *MdCHS* Genes in Combination With Chromatin State

Although the co-expression networks of *MdCHS1*, *MdCHS2*, and *MdCHS3* are highly similar, they still have some differences in that each network has its own specific genes. The expression levels of the three *MdCHS* genes in immature fruits were significantly higher than those in mature fruits, and in the young leaves, they were several hundred times higher than in the old leaves, indicating that *MdCHS* mainly functions in immature tissues. It can be seen that there are differences in the expression levels of the three *CHS* genes during fruit ripening, where the activity of *MdCHS2* is higher than that of *MdCHS1* and *MdCHS3* (**Supplementary Figure 10**). Interestingly, the order of the three *MdCHS* genes in terms of decrease in expression level was not synchronized at 5 weeks after full bloom (*MdCHS2* preceded *MdCHS3*, which preceded *MdCHS1*) (**Supplementary Figure 10**). Evolutionary analysis with MEGA6 revealed that *MdCHS2* and *MdCHS3* are on the same branch as *PbrCHS* of Chinese pear, while *MdCHS1* and *PcoCHS* of European pear are on the same branch, which also indicated that there are some differences in the functions of the three *MdCHS* genes (**Supplementary Figure 11**). By analyzing their expression patterns and evolutionary relationships, it can be seen that the three *MdCHS* genes have some differences. However, whether these differences are related to their chromatin environments remains unknown.

**Figure 4:**
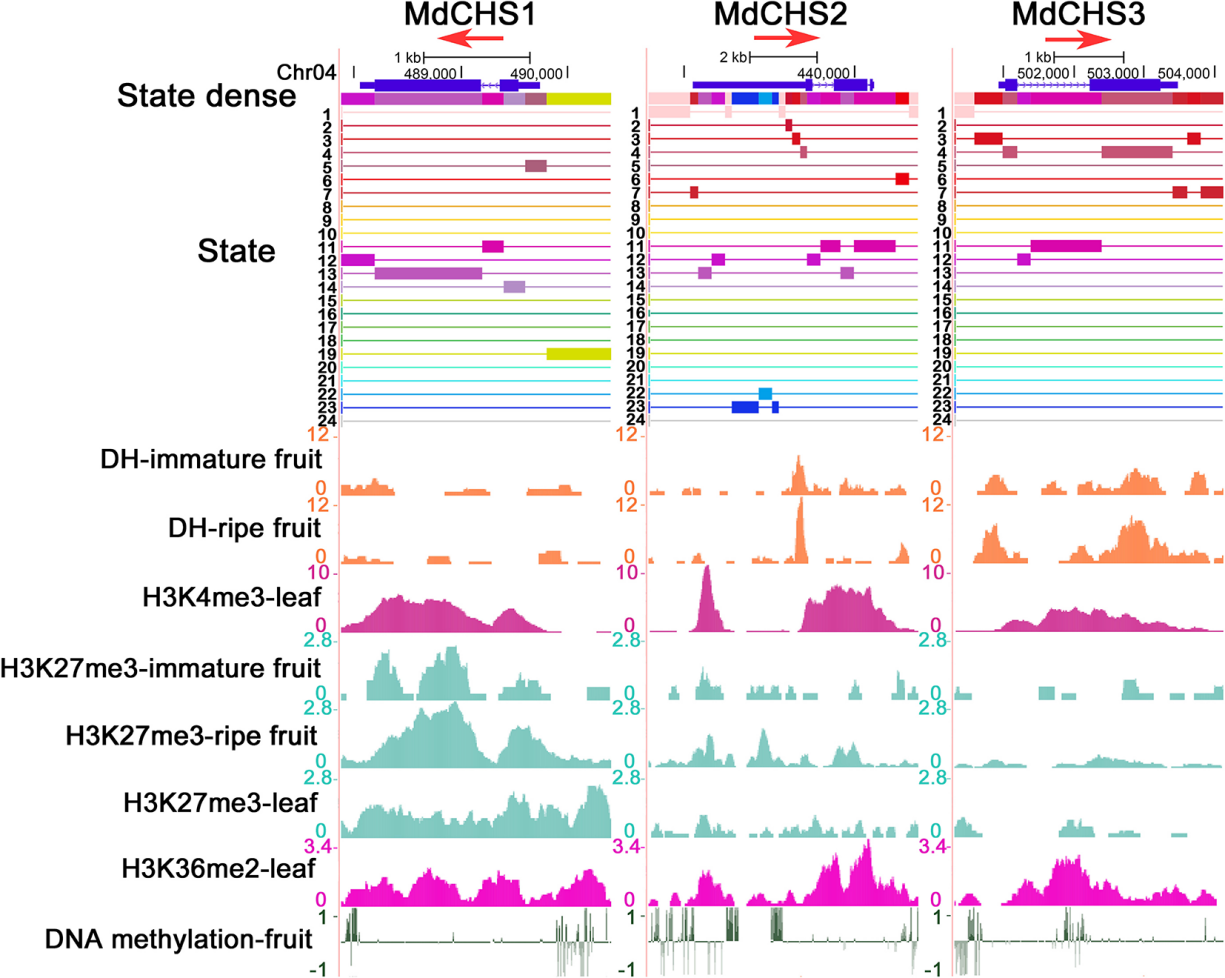
Chromatin state analysis of three *MdCHS* genes. Associated states and epigenetic marks of *MdCHS1*, *MdCHS2*, and *MdCHS3* in the UCSC genome browser. The red arrow represents the direction of gene transcription.

We further combined epigenetic markers to observe the chromatin states of these three genes. The gene body regions of the three *MdCHS* genes are mainly in warm color because they have higher DHSs and/or H3K4me3 modification levels. However, there are also differences in their chromatin states, in which the upstream of TSS region of *MdCHS1* is marked in green (state 19) for H3K27me3 and H3K36me2 and the 5' UTR region of *MdCHS2* is marked in blue (state 22 and state 23) for DNA methylation, implying that differences in chromatin states may contribute to differences in the transcription levels of these three *MdCHS* genes (**Figure 4**). Therefore, chromatin state analysis can be used to reflect the chromatin environment of genes and assist in the analysis of gene expression activity.

## Discussion

With the development of sequencing technologies, research on apple has entered the era of big data. How to efficiently analyze and parse sets of multi-dimensional and complex omics data is a key issue. We constructed an online analysis platform, AppleMDO, for apple functional genomic data mining and gene functional identification based on the integration of multi-dimensional omics datasets, including genomic, transcriptomic, and epigenomic datasets.

A global network and tissue-preferential network were constructed in AppleMDO, which was necessary for analyzing the differences and similarities of the two types of networks. The global network, namely, the condition-independent network, covers as many different tissues and stresses as possible and reflects the most common co-expression relationships between genes. The conditional network has a certain degree of specificity because it discards interfering factors. With the accumulation of transcriptomic datasets, we can build various, more sensitive conditional networks, for example, networks based on single-cell RNA sequencing.

Organisms are complex regulatory systems, and there are inevitably some limitations to using a single omics dataset to explore functional regulation. Therefore, we hope to combine multiple omics methods to analyze biological processes. In addition to co-expression networks, protein–protein interaction networks are also provided in AppleMDO. For example, after adding protein–protein interaction networks to the co-expression networks of *CHS1*, *CHS2*, and *CHS3*, we found that some additional genes were also related to anthocyanin biosynthesis, such as *CHI*, *DFR*, and *KMD3* (**Supplementary Figure 12**). At the same time, the chromatin states at the epigenetic level can also be combined with a co-expression network. For example, the six genes co-expressed with *SnRK1.1* (SNF1-related kinase 1) (**Supplementary Figure 13A**), which is involved in sucrose-induced anthocyanin accumulation (Liu et al., 2017), have similar chromatin states. The gene body area is yellow, and the promoter regions are red, indicating that these co-expression genes are similar in terms of chromatin (**Supplementary Figure 13B)**.

Considering the differences between varieties, we used only the “Golden Delicious” apple cultivar to construct the co-expression network and identify chromatin states. In fact, we also surveyed the data of other varieties and found that the “Golden Delicious” apple cultivar accounts for the majority of apple high-throughput-omics datasets available to the public, and other varieties have either too-small datasets or poor sample types. The complete genome sequence of the apple cultivar “Hanfu” was obtained in 2019 (Zhang et al., 2019), and its transcriptome and epigenomic datasets will accumulate rapidly in the near future. With the accumulation of subsequent data, we could also construct networks and define chromatin states for other varieties and carry out comparative analysis between varieties.

In our study, several datasets produced from different techniques, experiments, and stages were combined and integrated to construct a co-expression network. Thus, the heterogeneity of datasets is a key factor to be considered. First, in the early stage of data processing, cluster analysis was performed on all datasets to remove outliers (**Supplementary Figure 1**). Second, the goal was mainly to reflect the correlation of expression trends between genes rather than to select genes differentially expressed between samples using FPKM values in AppleMDO. Third, we observed the distribution of FPKM values for RNA-seq datasets from 10 different platforms and discovered that the distributions of FPKM values were similar among those 10 platforms, with similar median values, indicating that these datasets are comparable. In addition, our laboratory has published some databases that use the same method to process transcriptomic datasets in order to construct co-expression networks (Tian et al., 2017; You et al., 2017). According to our previous research, the co-expression network constructed by this method works well.

We assigned chromatin to different states based on epigenetic marks and considered different types of epigenetic marks as much as possible, including activation marks and inhibition marks. However, epigenetic mark data for apple available to the public are still limited, such as a lack of H3K9me2, histone variants, and transcription factors, and the tissue diversity and conditions of these datasets are relatively poor. Because epigenetic marks differ among tissues, developmental stages, and stress treatments, constant updates will be needed as datasets accumulate.

In summary, AppleMDO was established. Users can submit locus IDs to quickly search for co-expression networks, functional modules, chromatin states, and enriched epigenetic marks. For the gene list in the search results, gene expression profiling analysis and functional enrichment analysis tools are provided to systematically extract biological themes from gene lists. Furthermore, the basic structural and functional annotations of each gene can be obtained, such as the gene family, KEGG terms, GO terms, orthologues in 13 other species, and Pfam domains. In addition, some functional support toolkits are also provided, such as GO analysis, blast, motif analysis, ID conversion, sequence extraction, and the UCSC genome browser. We hope that AppleMDO will benefit apple research communities and serve as a reference for other fruit species.

## Data Availability Statement

This website (http://bioinformatics.cau.edu.cn/AppleMDO/) is free and open to all users, and there is no login requirement.

## Author Contributions

LD performed data collection, data analysis, and database construction. YL helped to define the chromatin states. JY helped to construct the web server. TT and JS helped to prepare the manuscripts. XM supported the server maintaining and database administration. WX provided the application of the co-expression network and some key functional module identification. ZS and WX supervised the project. All authors read and approved the final manuscript.

## Conflict of Interest

The authors declare that the research was conducted in the absence of any commercial or financial relationships that could be construed as a potential conflict of interest.
