# Supplementary material for: AppleMDO: A Multi-Dimensional Omics Database for Apple Co-Expression Networks and Chromatin States
Source: Front Plant Sci. 2019 Oct 22;10:1333. doi: 10.3389/fpls.2019.01333 (PMC6817610; doi:10.3389/fpls.2019.01333)
Supplement: Supplementary file 1 [file DataSheet_1.pdf]

Supplementary Figures

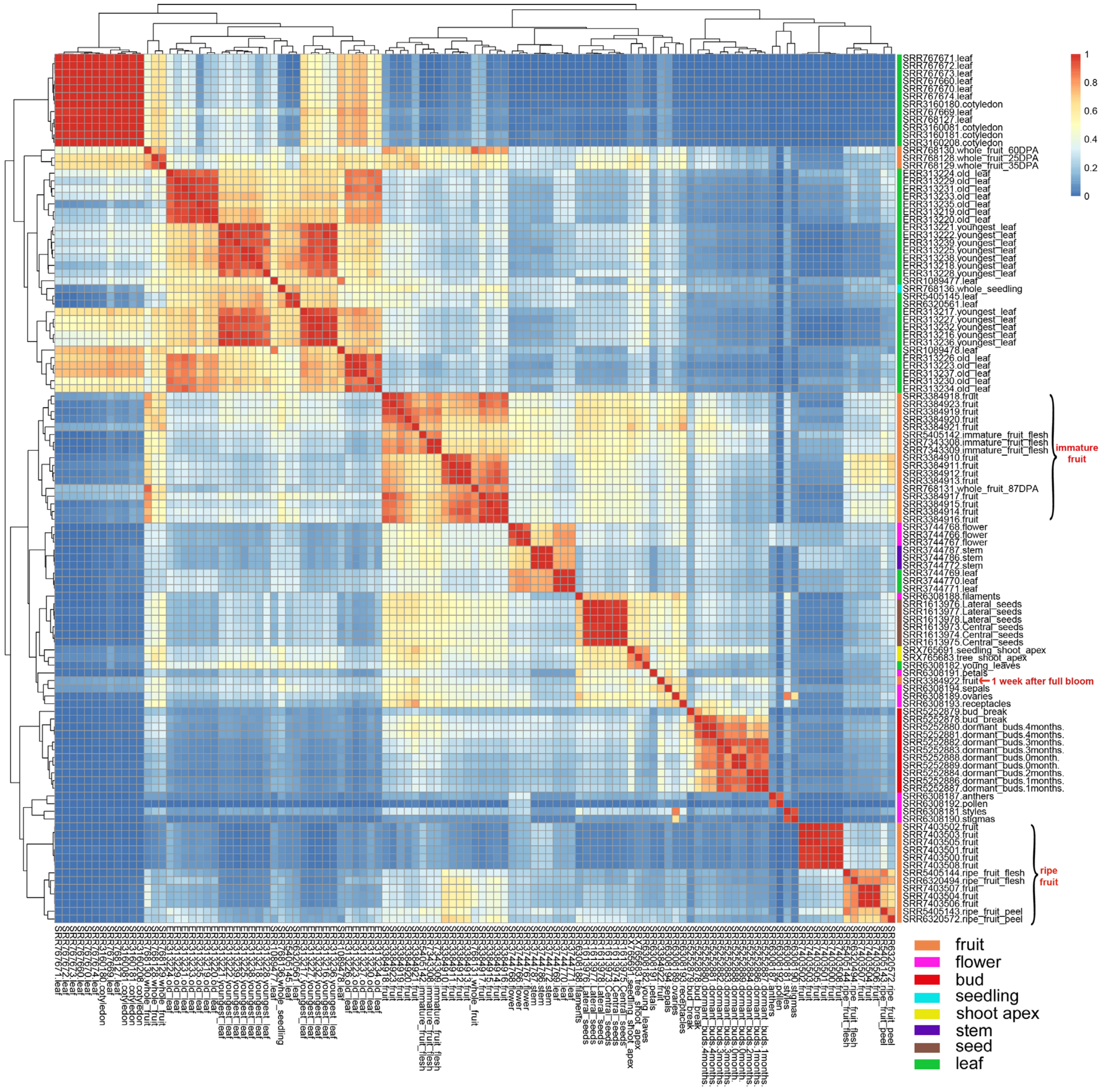

Supplementary Figure 1. Cluster analysis diagram for all RNA-seq datasets created by using pheatmap. The redder the colour is, the stronger the correlation between the two samples is. The bluer the colour is, the weaker the correlation between the two samples is.

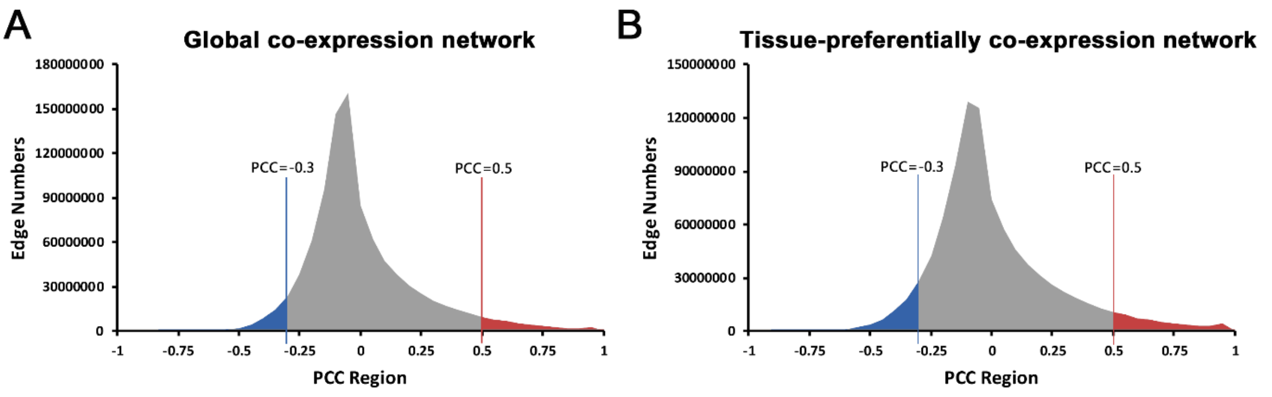

Supplementary Figure 2. The PCC distribution of all gene pairs in the co-expression network. The blue region (PCC ≤ -0.3) represents the lowest 5% of gene pairs and the red region (PCC ≥ 0.5) represents the highest 5% of gene pairs based on their PCC values in the global (A) and tissue-preferential (B) co-expression networks.

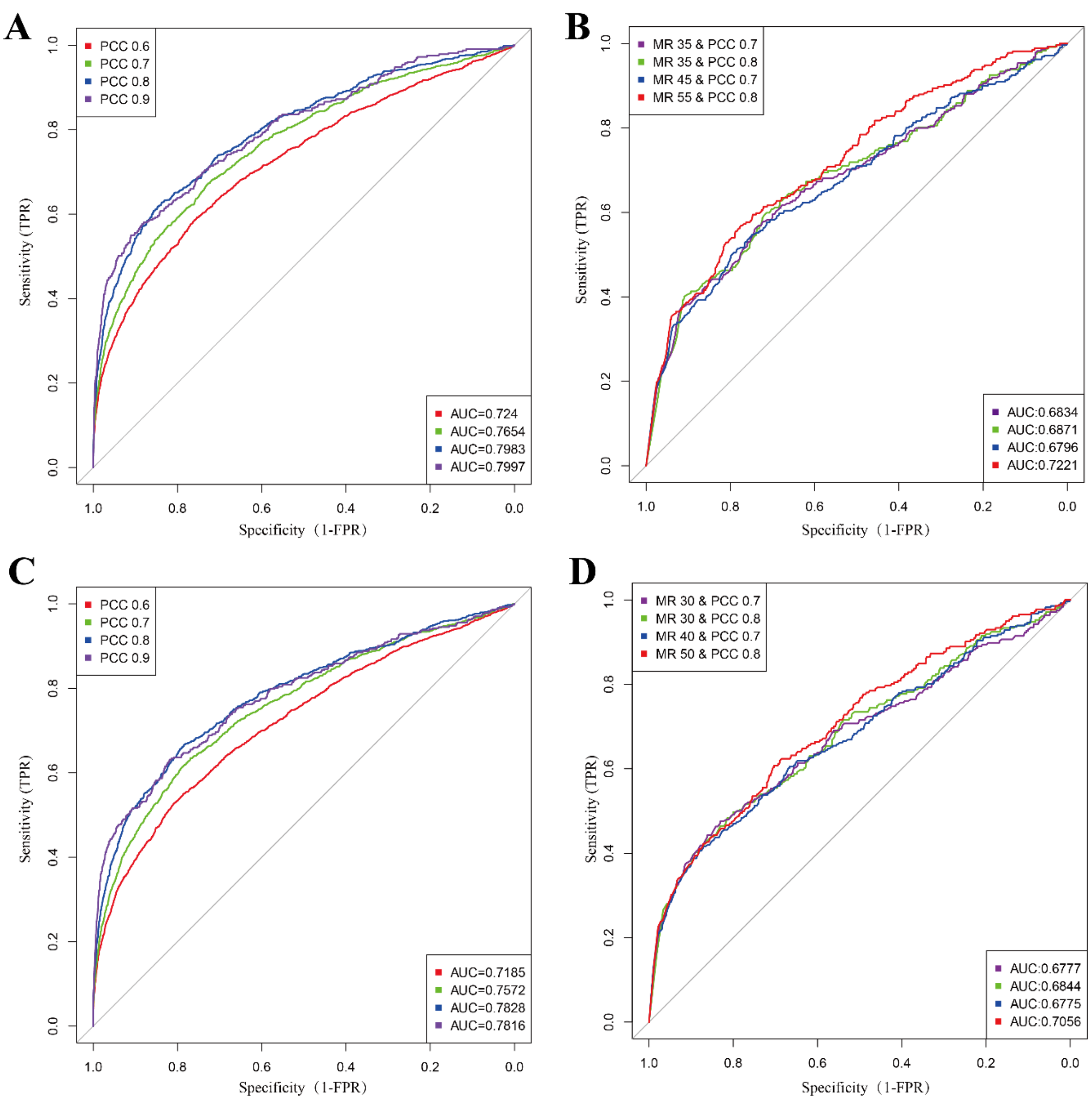

Supplementary Figure 3. Selection of PCC and MR thresholds for co-expression networks. (A) A plot of the true-positive rate [TP/(TP + FN)] against the false-positive rate [TN/(FP + TN)] of the possible global co-expression networks with different PCC thresholds (PCC ≥ 0.6, PCC ≥ 0.7, PCC ≥ 0.8 and PCC ≥ 0.9). (B) A plot of the true-positive rate [TP/(TP + FN)] against the false-positive rate [TN/(FP + TN)] of the possible global co-expression networks with different MR and PCC thresholds (MR<35 and PCC ≥ 0.7, MR ≤ 35 and PCC ≥ 0.8, MR ≤ 45 and PCC ≥ 0.7 and MR ≤ 55 and PCC ≥ 0.8). (C) A plot of the true-positive rate [TP/(TP + FN)] against the false-positive rate [TN/(FP + TN)] of the possible tissue-preferential co-expression networks with different PCC thresholds (PCC ≥ 0.6, PCC ≥ 0.7, PCC ≥ 0.8 and PCC ≥ 0.9). (D) A plot of the true-positive rate [TP/(TP + FN)] against the false-positive rate [TN/(FP + TN)] of the possible tissue-preferential co-expression networks with different MR and PCC thresholds (MR ≤ 30 and PCC ≥ 0.7, MR ≤ 30 and PCC ≥ 0.8, MR ≤ 40 and PCC ≥ 0.7 and MR ≤ 50 and PCC ≥ 0.8).

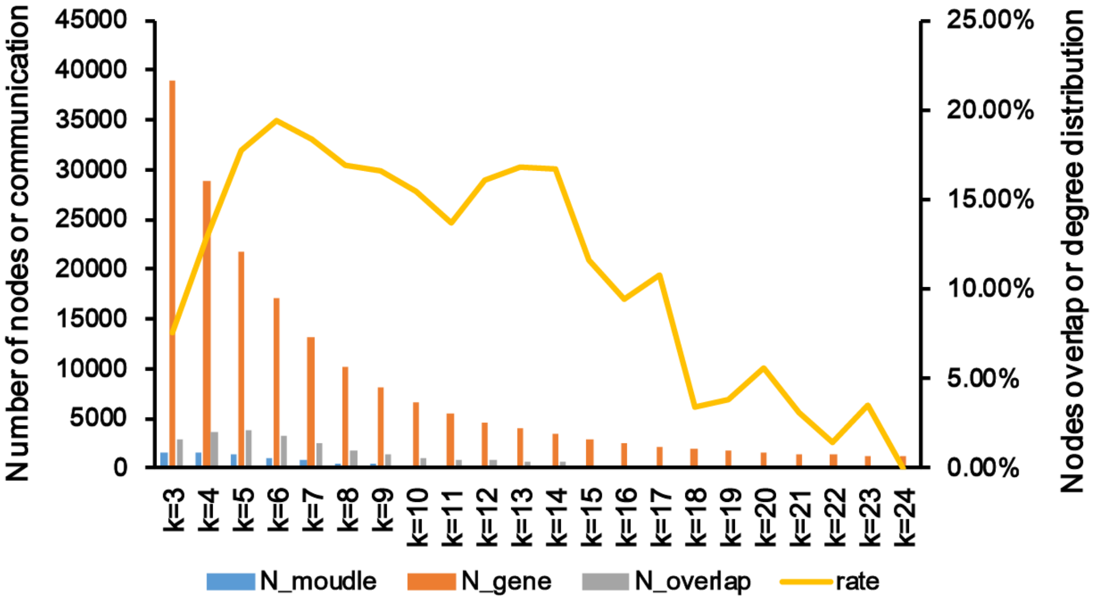

Supplementary Figure 4. The selection of parameter k values for the modules. CFinder software was applied to calculate communities of different k-clique sizes (from k = 3 to k =34). When k = 6, there is a larger number of functional modules (communities), more gene coverage and more community overlap.

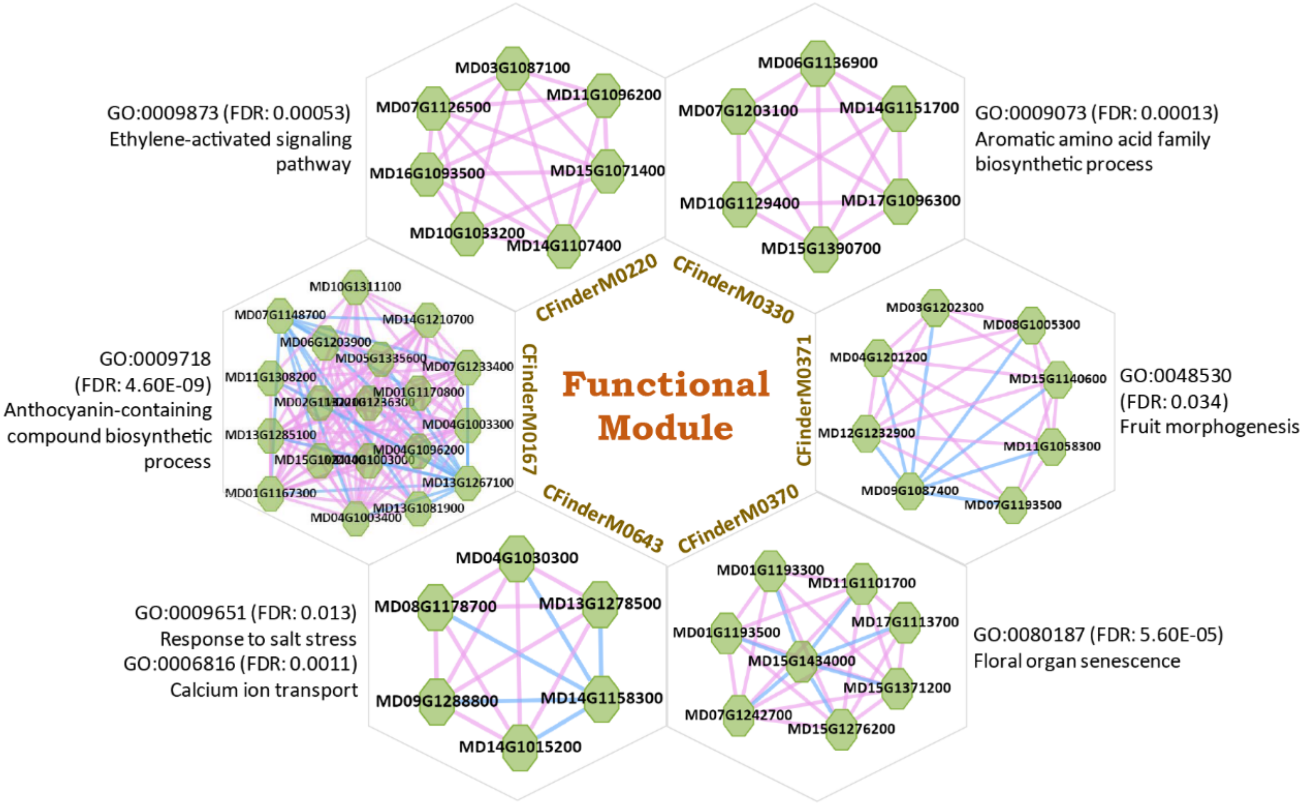

Supplementary Figure 5. A combination of functional regulatory modules related to apple agronomic traits obtained through module prediction. The functions of the modules were predicted by integrating annotations such as GO terms, gene families, and KEGG annotations, and non-significant entries were filtered by Fisher's test and multiple hypothesis testing (FDR $\leq$ 0.05).

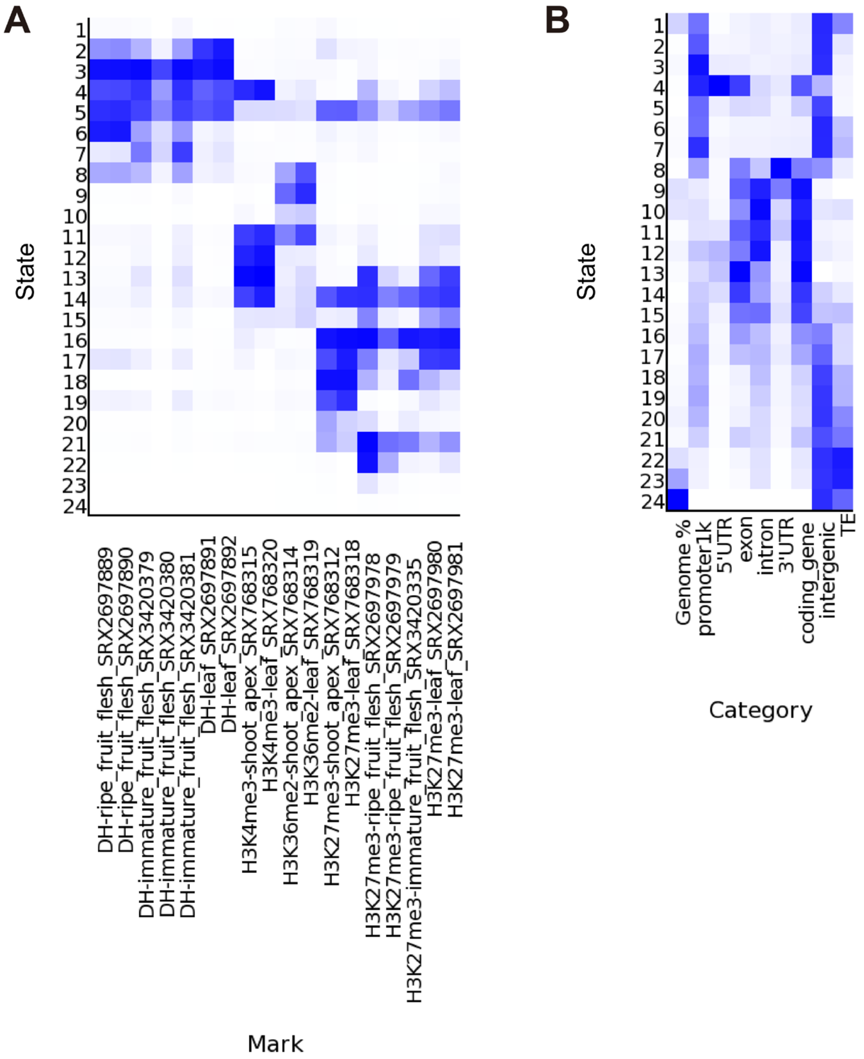

Supplementary Figure 6. Heatmaps of emission parameters and fold enrichment of genomic elements. (A) The distribution of the epigenetic marks among 24 chromatin states. The X-axis represents the epigenetic marks, and the Y-axis represents 24 chromatin states. (B) Fold enrichment of 24 chromatin states among different feature regions on genome. The X-axis represents different feature regions on genome, and the Y-axis represents 24 chromatin states.

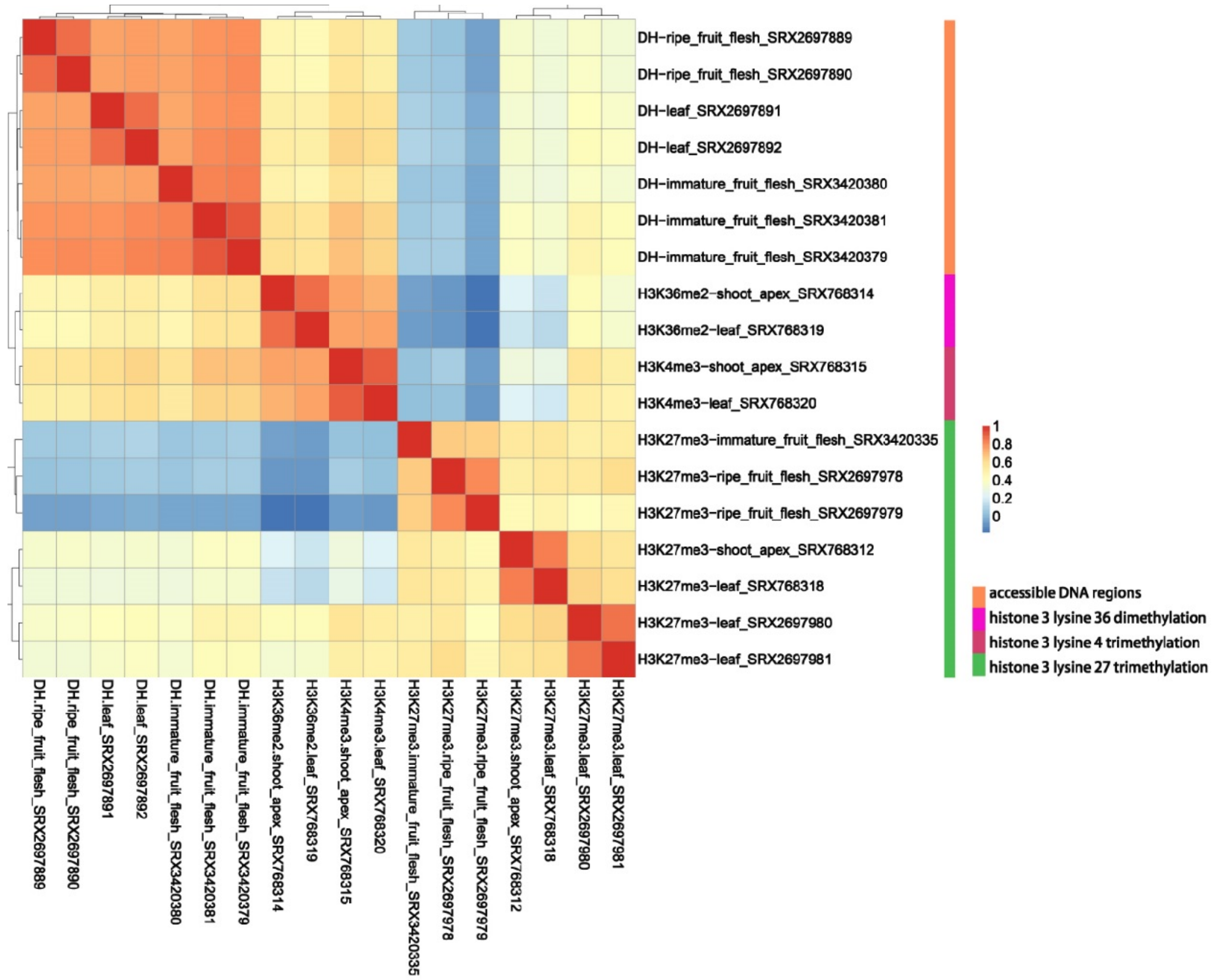

Supplementary Figure 7. All epigenomic datasets were clustered using the plotCorrelation in deepTools software.

State	Preferential epigenetic marks	Preferential location	Color
state 1	weak accessible DNA	intergenic, promoter,TE	
state 2	accessible DNA	promoter, intergenic	
state 3	accessible DNA	promoter, intergenic	
state 4	accessible DNA, H3K4me3, weak H3K27me3	promoter, 5'UTR, exon, intergenic	
state 5	accessible DNA, H3K27me3, weak H3K4me3, weak H3K36me2	intergenic, promoter	
state 6	accessible DNA	intergenic, promoter	
state 7	accessible DNA	intergenic, promoter	
state 8	H3K36me2, accessible DNA	3'UTR, exon, intergenic, promoter	
state 9	H3K36me2	intron, exon, 3'UTR	
state 10	weak H3K36me2	intron	
state 11	H3K4me3, H3K36me2, weak H3K27me3	intron, exon	
state 12	H3K4me3, weak H3K27me3, weak accessible DNA	intron	
state 13	H3K4me3, H3K27me3, weak accessible DNA	exon	
state 14	H3K4me3, H3K27me3, weak accessible DNA	exon	
state 15	H3K27me3, weak H3K36me2, weak H3K4me3	intron, exon	
state 16	H3K27me3	exon, intergenic, promoter, intron	
state 17	H3K27me3, weak accessible DNA	intergenic, promoter, intron, exon	
state 18	H3K27me3	intergenic, TE, promoter	
state 19	H3K27me3, accessible DNA	intergenic, promoter, TE	
state 20	weak H3K27me3	intergenic, TE, promoter	
state 21	H3K27me3	intergenic, TE	
state 22	H3K27me3	TE, intergenic	
state 23	weak H3K27me3	TE, intergenic	
state 24	rare signal	intergenic, TE	

Supplementary Figure 8. Predicted chromatin states marked in different colours. The states with activation of transcription are marked in warm colours, whereas the states with repression of transcription are marked in cool colours.

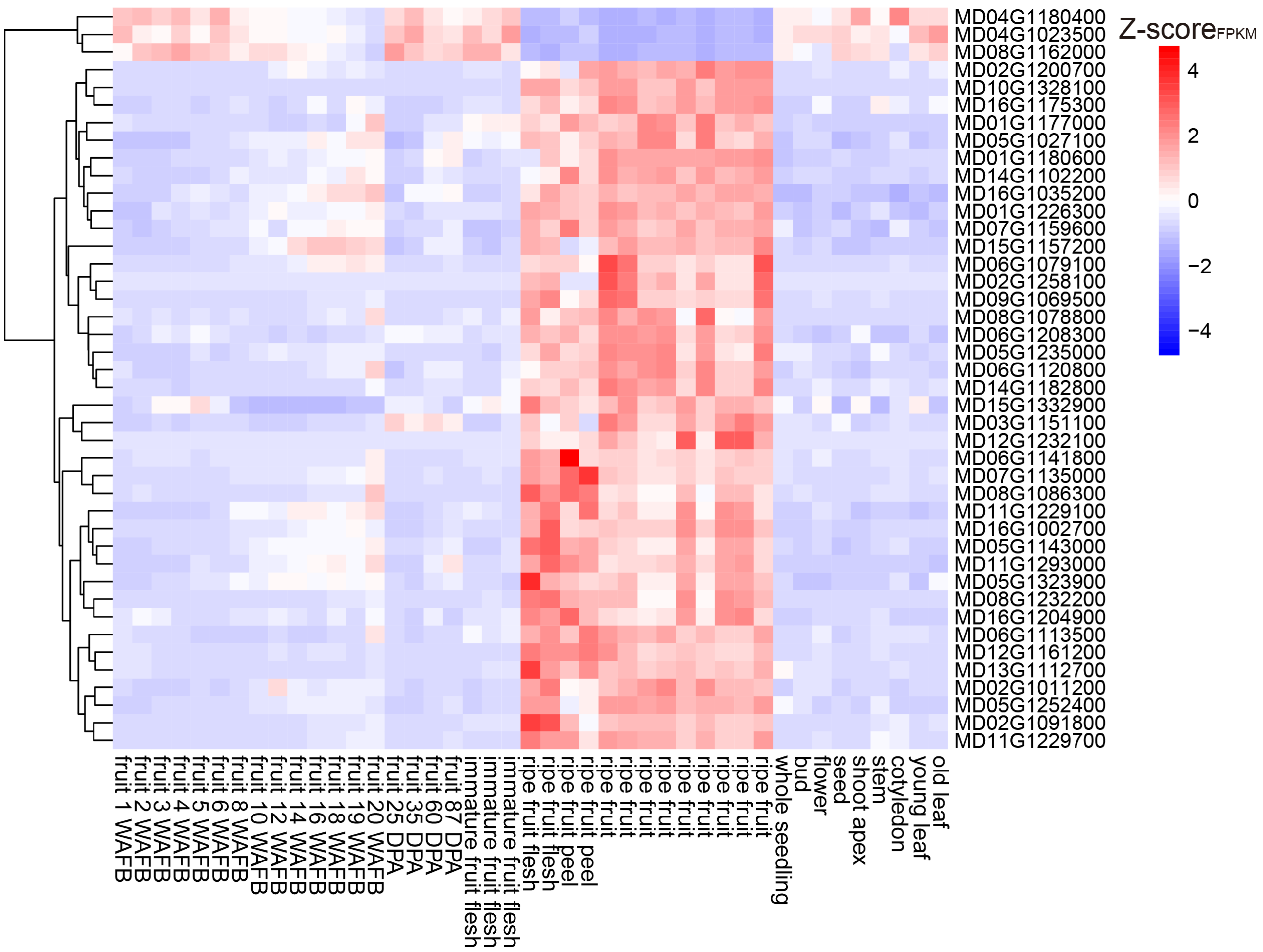

Supplementary Figure 9. Hierarchical cluster analysis of the expression values of *ACO1* co-expressed genes based on Z-score tests.

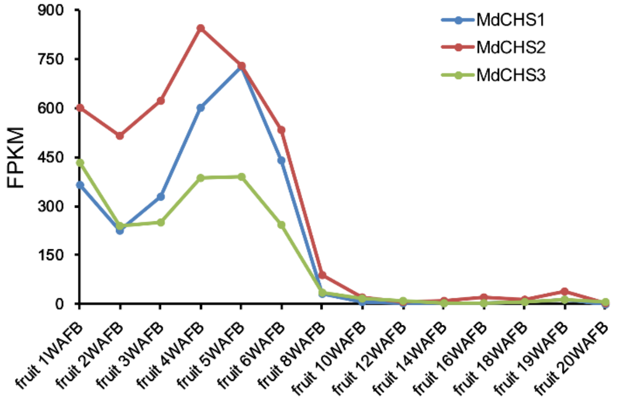

Supplementary Figure 10. The expression level of three *MdCHS* genes in fruit flesh at different weeks after full bloom.

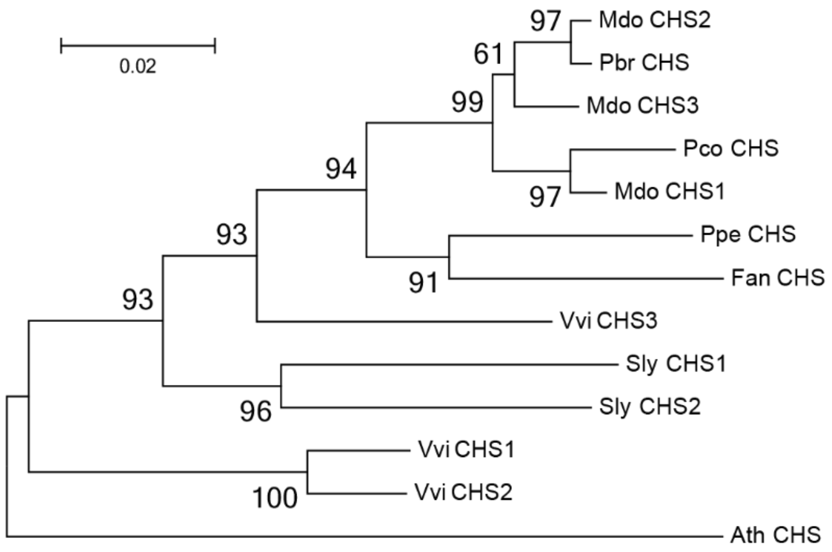

Supplementary Figure 11. Evolutionary analysis of *CHS* in fruits with MEGA6. *MdoCHS1* (MD04G1003300), *MdoCHS2* (MD04G1003000), *MdoCHS3* (MD04G1003400), *PbrCHS* (*Pyrus bretschneideri*; KF148032), *PpeCHS* (*Prunus persica*; HM543568.1), *PcoCHS* (*Pyrus communis*; DQ901397), *FanCHS-2* (*Fragaria ananassa*; AB201756), *SlyCHS1* (*Solanum lycopersicum*; X55194.1; Solyc09g091510.2), *SlyCHS2* (*Solanum lycopersicum*; X55195.1; Solyc05g053550.2), *VviCHS1* (*Vitis vinifera*; AB015872), *VviCHS2* (*Vitis vinifera*; AB066275), *VviCHS3* (*Vitis vinifera*; AB066274) and *AthCHS* (*A. thaliana*; AT5G13930.1)

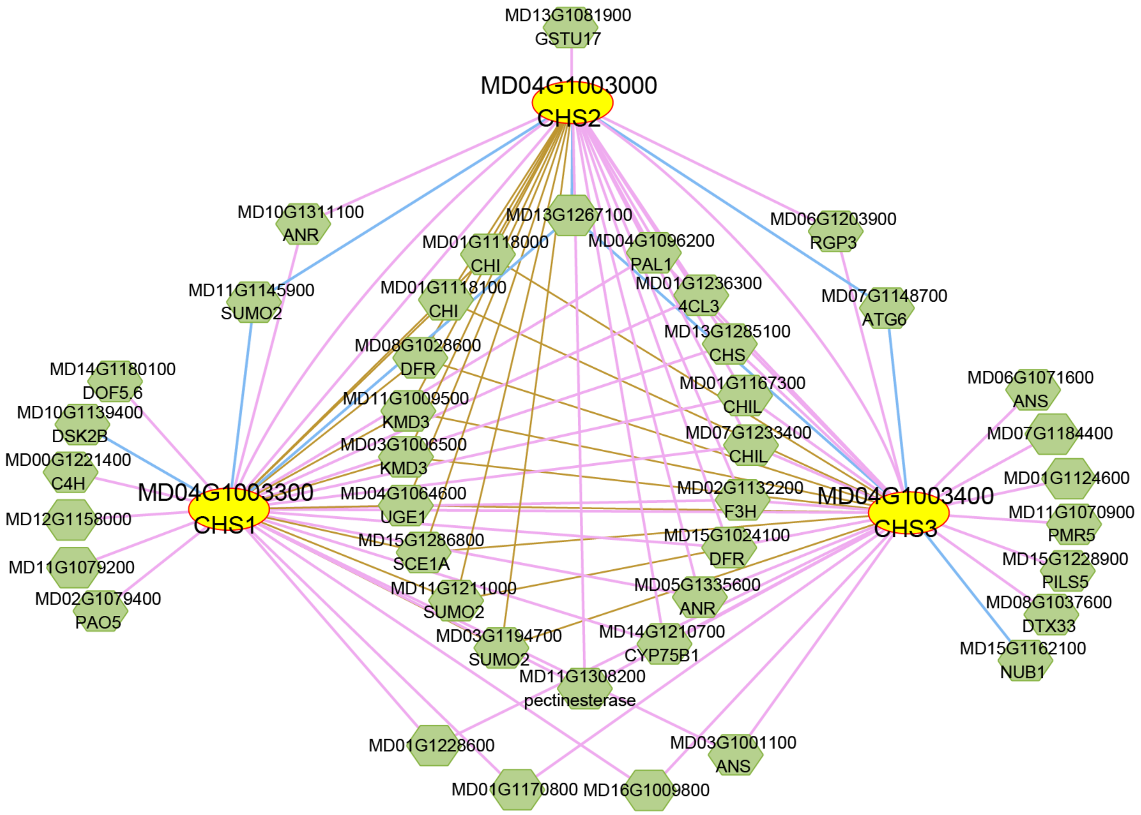

Supplementary Figure 12. Network of *CHS* combining co-expression and protein-protein interactions. The yellow circles are three *CHS* genes, and the green polygons are genes co-expressed with *MdCHS*. The pink line indicates positive co-expression relationship with *MdCHS*, the blue line indicates negative co-expression relationship with *MdCHS*, and the brown line indicates protein-protein interaction relationship.

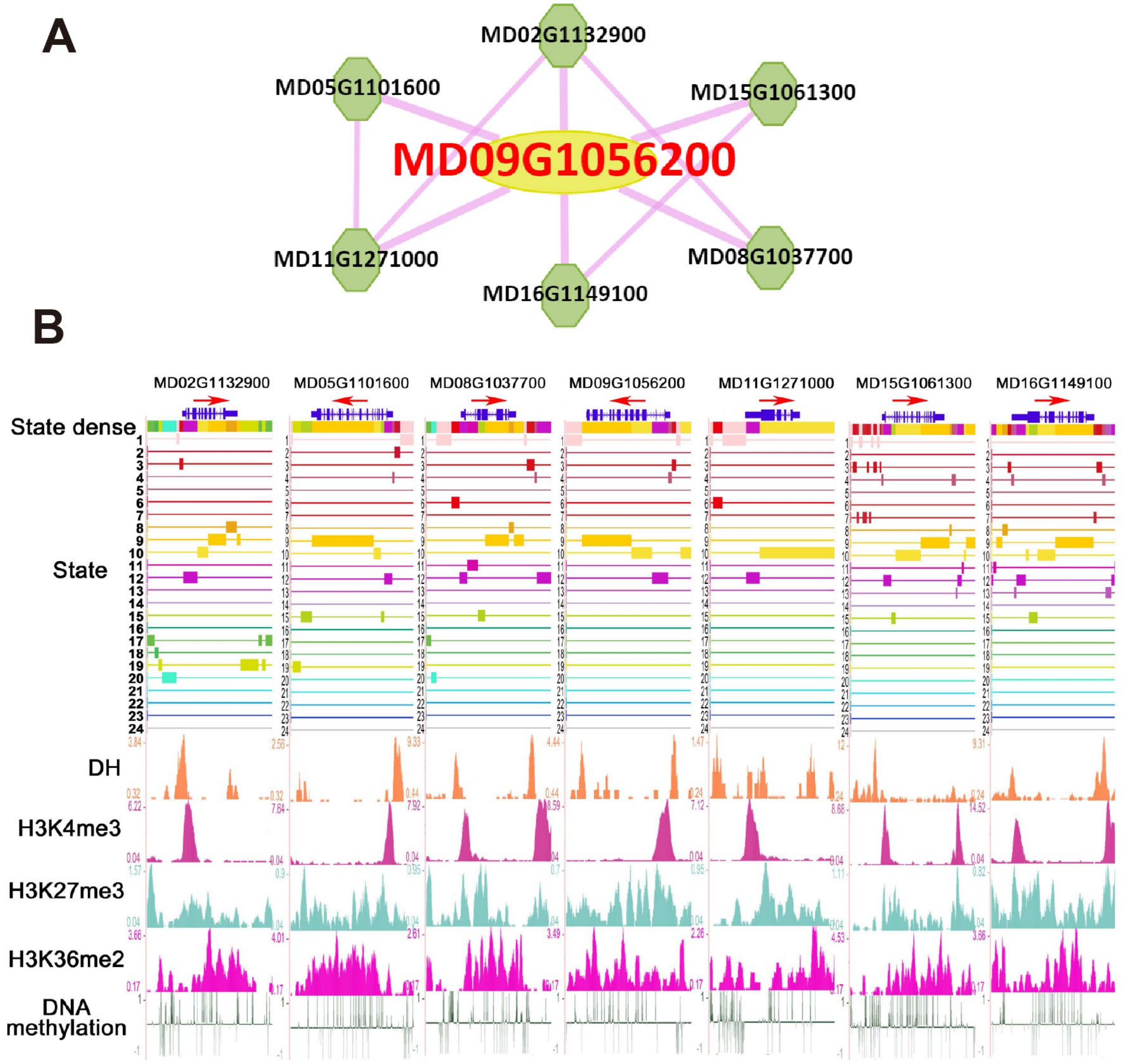

Supplementary Figure 13. Analysis of genes co-expressed with *MdSnRK1.1* in combination with chromatin states. (A) The co-expression network of SnRK1.1. The yellow circle is the *MdSnRK1.1* gene, and the green polygons are genes co-expressed with *MdSnRK1.1*. The pink line indicates positive co-expression with *MdSnRK1.1*. (B) Associated states and epigenetic marks of *MdSnRK1.1* in the UCSC genome browser. The red arrow represents the direction of gene transcription.
