# Supplementary material for: AppleMDO: A Multi-Dimensional Omics Database for Apple Co-Expression Networks and Chromatin States
Source: Front Plant Sci. 2019 Oct 22;10:1333. doi: 10.3389/fpls.2019.01333 (PMC6817610; doi:10.3389/fpls.2019.01333)
Supplement: Supplementary file 2 [file Table_1.doc]

**Supplementary Table 1.** Co-expression network results.

| **Network** | **Global** | **Conditional** |
| --- | --- | --- |
| Samples | 112 RNA-seq datasets | 81 RNA-seq datasets |
| Tissues | seedling, fruit, fruit flesh, fruit peel, seeds, flower, stigmas, styles, filaments, anthers, pollen, petals, sepals, receptacles, ovaries, buds, shoot apex, stem, leaf | seedling, fruit, fruit flesh, fruit peel, seeds, flower, stigmas, styles, filaments, anthers, pollen, petals, sepals, receptacles, ovaries, buds, shoot apex, stem, leaf |
| Stress treatments | infected by pathogen | no |
| PCC range (positive) | [0.8, 1] | [0.8, 1] |
| PCC range (negative) | [-1, -0.6] | [-1, -0.65] |
| MR range | [1, 55] | [1, 50] |
| Nodes | 43,862 (97.2%) | 42,991 (95.3%) |
| Positive edges | 553,780 | 478,615 |
| Negative edges | 206,082 | 204,650 |
